# The Changing Global Epidemiology of Re-emerging Human Monkeypox Virus Infection: A Systematic Review

**DOI:** 10.7759/cureus.45123

**Published:** 2023-09-12

**Authors:** Sunder Sham, FNU Sapna, FNU Anjali, Sanjay Kumar, Vivek Podder, Soumya Jaladi, Ahmed Bendari, Reham Al-Refai, Manal M Baloch, Mohammed Abdelwahed, Nfn Kiran, Saroja Devi Geetha, Hansini Laharwani

**Affiliations:** 1 Pathology and Laboratory Medicine, Lenox Hill Hospital, Northwell Health, New York, USA; 2 Pathology and Laboratory Medicine, Albert Einstein College of Medicine, Bronx, USA; 3 Internal Medicine, Sakhi Baba General Hospital, Sukkur, PAK; 4 Gastroentrology, Bahria University of Health Sciences, Karachi, PAK; 5 General Medicine, Tairunnessa Memorial Medical College and Hospital, Gazipur, BGD; 6 Pathology and Laboratory Medicine, University of Louisville, Louisville, USA; 7 Internal Medicine, Bahria University of Health Sciences, Karachi, PAK; 8 Pathology and Laboratory Medicine, Donald and Barbara Zucker School of Medicine at Hofstra/Northwell, Uniondale, USA; 9 Pathology and Laboratory Medicine, Staten Island University Hospital, Staten Island, USA; 10 Pathology and Laboratory Medicine, Washington University School of Medicine, St. Louis, USA

**Keywords:** vaccine, outbreak and pandemic, infections, monkeypox virus, monkeypox outbreak

## Abstract

Human monkeypox virus (MPVX) infection represents an emerging zoonotic disease caused by an orthopoxvirus, resulting in a condition reminiscent of smallpox. More recent developments have witnessed a notable surge in global MPVX outbreaks, eliciting significant concerns. We aimed to investigate the epidemiological factors of the emerging human monkeypox virus infection, including the number of suspected, confirmed, and fatal cases, as well as the risk factors for contracting monkeypox infection. We performed a systematic review of peer-reviewed literature by following the Preferred Reporting Items for Systematic Reviews and Meta-Analyses (PRISMA) guidelines. An electronic database search (PubMed, Wiley Online Library, and Science Direct) was undertaken. For monkeypox-related studies, we included 25 peer-reviewed articles from 2018 and 2022, and data were extracted on the current evidence on the cases and the risk factors for MPVX infection, to develop public health advisories. Our reports show a rapid rise of MPVX cases in the highly endemic African regions after the 1970s, spread to other countries, and an increase in the median age from young children to young adults. The cessation of smallpox vaccination might have been one of the factors responsible for these findings. As of 2022, the genomic sequences of ten MPVX strains associated with the recent countrywide outbreak have been determined. While the West African Clade has been primarily implicated in the recent viral surge, data were insufficient to determine which mutation contributed to increased transmissibility. In the Democratic Republic of the Congo (DRC), sleeping on the floor was significantly associated with contracting MPVX, while eating or processing of animal foods was not a significant risk factor. In the United States, cleaning the cages and bedding of sick animals, touching infected animals, and daily exposure to sick animals were associated with an increased probability of contracting the MPVX infection. Recent global outbreaks and the rising incidence of MPVX infections among young adults in the endemic zones might be a result of the cessation of the smallpox vaccine. The increased risk associated with exposure to sick animals or sleeping on the floor suggests high infectivity from animal excretions. Increasing awareness, strict surveillance, and contact tracing can help contain global outbreaks. The ring vaccination approach for exposed individuals is another potential disease containment strategy. Future studies should investigate measures for rapid laboratory diagnosis, maintaining lab safety, and transmissibility.

## Introduction and background

As the world adjusts to the shock of the coronavirus-2019 (COVID-19) pandemic and lessons are being learned from the recent failures to control the global disease outbreak, new infectious disease threats, such as the human monkeypox virus (MPVX) infection continue to emerge [1]. Since May 7, 2022, the unprecedented and unexpected MPVX outbreaks across Europe, the US, and Australia, which have continuously been reported from 12 World Health Organization (WHO) member states across three WHO regions, have surprised the health authorities worldwide [1]. MPVX infection, an emerging zoonotic disease caused by an orthopoxvirus, results in clinically milder smallpox-like symptoms. With the eradication of smallpox in 1980 and the subsequent cessation of the smallpox vaccination, MPVX has emerged as the most concerning orthopoxvirus threat to public health [2]. In 1970, MPVX was first identified in a nine-month-old boy in the Democratic Republic of the Congo (DRC), where smallpox had been eliminated in 1968. Before April 2022, MPVX cases were endemic and seldom reported outside the African regions, where it primarily occurred in Central and West Africa, often in proximity to tropical rainforests [2-4]. Epidemiologic investigations could not establish a travel link to the reported increase in cases within endemic areas. Animal hosts of this virus include a range of rodents and non-human primates that transmit the virus to humans after an incubation period of five to 21 days [1-3].

MPVX is an enveloped, double-stranded DNA virus of the Orthopoxvirus genus belonging to the Poxviridae family [4]. It has two distinct genetic clades, namely, the Central African (Congo Basin (CB)) clade and the West African (WA) clade [4]. Historically, the Congo Basin clade has caused more severe disease and was thought to be more transmissible. The geographical division between the two clades has so far been in Cameroon, the only country where both clades have been found [4,5]. The true burden of MPVX cases is unknown [6]. Since 2017, Nigeria has experienced a large outbreak with over 500 suspected and 200 confirmed cases with a 3% fatality ratio [7].

In 2003, the US experienced the first MPVX outbreak of 70 cases outside of Africa, which was linked to contact with infected pet prairie dogs [8]. These pets had been housed with Gambian pouched rats and dormice that had been imported into the country from Ghana [8]. Monkeypox has also been reported in travelers from Nigeria to Israel (2018), the United Kingdom (2018, 2019, 2021, and 2022), Singapore (2019), and the US (2021). In May 2022, multiple cases of monkeypox were identified in several non-endemic countries, as mentioned earlier [9]. MPVX infection has become a disease of global public health importance because it is spreading in epidemic proportions globally.

The recent increase in human MPVX cases across a wide geographic area, the potential for further spread, and the lack of reliable surveillance have heightened concerns about this emerging zoonotic disease. In November 2017, a collaborative consultation of the WHO and the Centers for Disease Control and Prevention (CDC) with global researchers, global health partners, ministries of health, and orthopoxvirus experts reviewed and discussed the recently detected human MPVX infections in African countries and also identified surveillance and other improvement measures [10]. Endemic human monkeypox has been reported from more countries in the past decade than during the last 40 years. Since 2016, confirmed cases of monkeypox have occurred in the Central African Republic, the Democratic Republic of the Congo, Liberia, Nigeria, Republic of the Congo, and Sierra Leone, and captive chimpanzees in Cameroon [11]. Many countries with endemic monkeypox lack recent experience and specific knowledge about the disease to detect cases, treat patients, and prevent the virus from spreading further. Specific improvements in surveillance capacity, laboratory diagnostics, and infection control measures are needed to mount an efficient response [12-19]. The recent global outbreaks of MPVX following the staggering COVID-19 pandemic have given scientists the impetus to investigate changes in epidemiology to develop disease mitigation and containment measures.

Therefore, we aimed to systematically review the current literature on the evolutionary epidemiology of emerging human MPVX infections and emphasize the number of suspected, confirmed, and fatal cases, as well as the risk factors associated with contracting monkeypox infections. 

This article was previously posted to the medRxiv preprint server on February 17, 2023. The preprint version is available with the following DOI: https://doi.org/10.1101/2022.12.09.22283261.

## Review

Methodology

Search Strategy

We performed a systematic review of peer-reviewed literature in accordance with the international standards for conducting and reporting systematic reviews, including the Preferred Reporting Items for Systematic Reviews and Meta-Analyses (PRISMA) guidelines. Three electronic databases, including MEDLINE (accessed using PubMed), the Online Willey Library, and Science Direct, were searched with no language restrictions. For monkeypox-related studies, we included articles from 2018 to 2022 for better comparison.

The search string used the following keywords in all three databases: “Orthopox virus” OR “Monkeypox virus” OR “Human Infection” and “Transmission of Virus” to search relevant articles. We aimed to explore how monkeypox epidemiology has evolved by including data related to the number of suspected, confirmed, and fatal cases. We also sought to explore the risk factors for contracting monkeypox infections. We ensured that all the data have information such as transmission, pathology, and evolution, as well as other features of the virus. Two independent researchers screened the titles and abstracts of the studies retrieved from the electronic search, and the relevant studies were evaluated in full text for eligibility.

Inclusion and Exclusion Criteria

Peer-reviewed articles with complete demographic information and complete medical records of monkeypox patients were included. Non-English studies, letters to editors, posters, case studies with incomplete information, non-human studies, modeling studies, case reports, and articles without full text were excluded from the review.

Data Extraction

All citations were imported into a bibliographic database, and duplicates were removed. Titles, abstracts, and subsequently, the full text of all articles were screened for eligibility. All the studies that had directly included transmission of MPVX, reported outbreaks, and signs and symptoms of MPVX were included in the review. The following information was extracted from each study: a) details of the study (study setting, year of publication, and study design); b) study population, sample size (male/female), and age in years; c) primary intervention(s) and control group; and d) transmission of MPVX. The further evaluation of selected data was conducted in two phases. Firstly, we selected the data based on the titles and abstracts. Secondly, the full text of the articles was examined and included if they satisfied the eligibility criteria for inclusion.

Results

**Figure 1:**
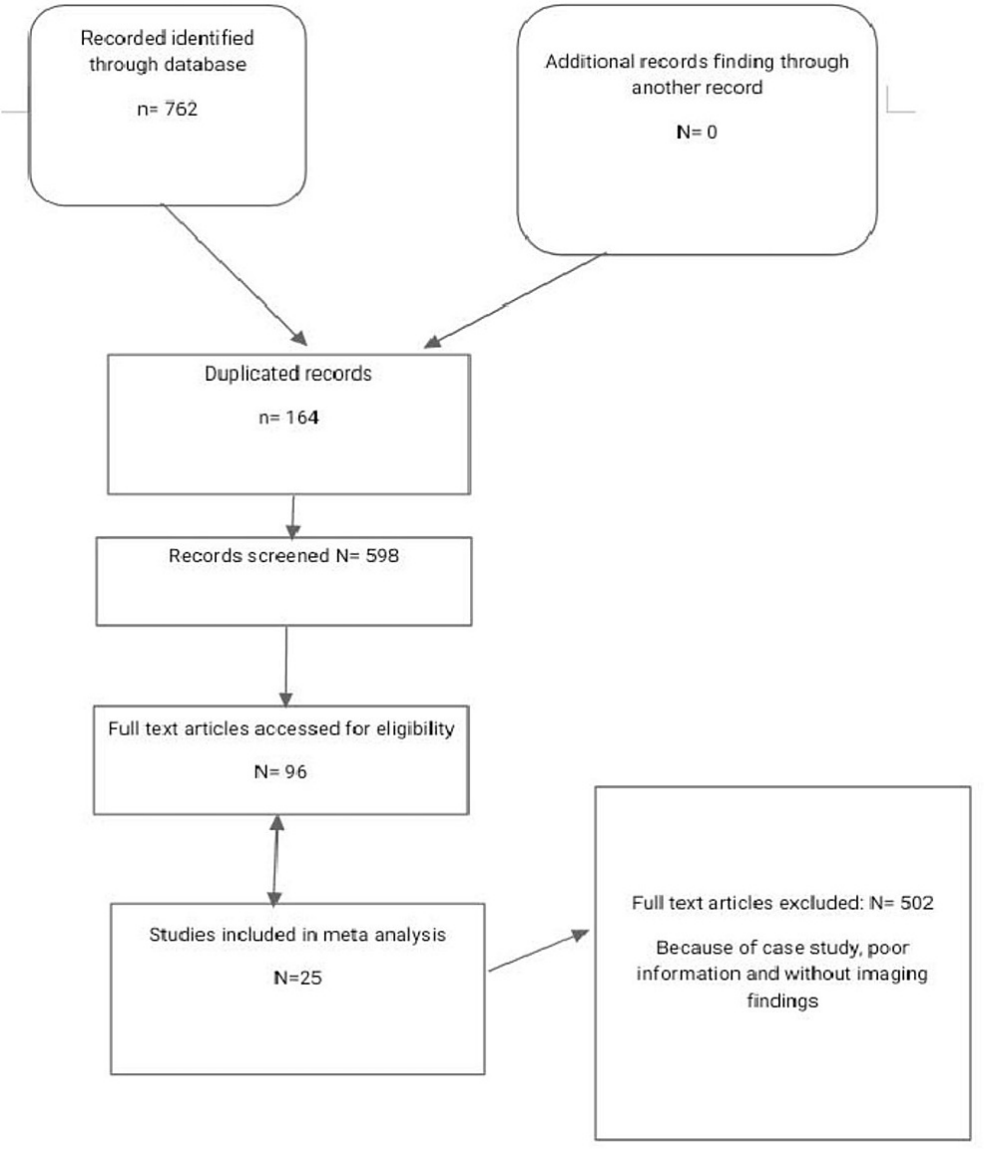
Inclusion criteria of selected studies according to PRISMA follow-up. PRISMA: Preferred Reporting Items for Systematic Reviews and Meta-Analyses.

The initial search strategy yielded a total of 762 articles, 164 of which were removed due to duplicated articles, and the remaining 598 articles were screened for full-text eligibility. On further screening, we omitted 502 articles with poor information. The remaining 96 articles were further screened for inclusion criteria and 25 articles that fulfilled the inclusion criteria and had adequate data were included. The PRISMA flowchart of the selection process for the systematic review is shown in Figure 1.

Number of Reports by Country

Monkeypox data from the DRC accounted for more than one-third of the eligible articles [2,4,8-11,13,14,17,18,20-23]. The remaining articles had monkeypox data from Gabon [5,7], Sierra Leone [3,20], Cameroon [4,6,23], CAR [4,12,15,24-28], ROC [13,16,19,29], Cote d’Ivoire [3,4], Nigeria [3,30], Liberia [3,31-34], and Pakistan [35]. Note: Two articles [3,4] described data for more than one country; therefore, the total number of articles per country exceeds 25.

Number of Cases by Country

We identified 25 peer-reviewed articles [1-12,15-24,28-30] with data on the number of confirmed cases, number of deaths, and case fatality rate (CFR) associated with human MPVX infection. Since the beginning of 2022, the continent has reported 4,667 cases (559 confirmed; 4,108 suspected) and 127 deaths (CFR: 2.8%) due to monkeypox infections from eight endemic Africa Union member states (MS): Benin (three suspected cases; three confirmed cases; zero deaths), Cameroon (29; 7; 2), CAR (17; 8; 2), Congo (14; 5; 3), DRC (2,775; 163; 110), Ghana (535; 84; 4), Liberia (31; 2; 0), and Nigeria (704; 277; 6); and four non-endemic MS: Egypt (0; 1; 0), Morocco (0; 3; 0), South Africa (0; 5; 0), and Sudan (0; 1; 0). This week, a total of 423 new cases (39 new confirmed cases; 384 new suspected cases) and three new deaths due to monkeypox infection were reported from Benin, Congo, Ghana, and Liberia (Table 1).

**Table 1:** Suspected, confirmed and fatal monkeypox cases by country and year. Each box denotes the number of probable cases, number of confirmed cases (n), number of deaths, and/or CFR (%). Where suspect cases were tested and found positive for another disease, these were subtracted from the suspect case total. CFR was calculated as the number of deaths out of the total suspected cases. *Available data on outbreaks and case numbers in DRC indicate that monkeypox is endemic, with more than 1000 suspected cases reported annually. †One death (n = 1) had an unclear cause, possibly linked to surgery or acute MPVX. Another case (n = 1) involved viral conjunctivitis after MPVX, causing corneal damage. An infant (n = 1) didn't fully recover and died six months later, reportedly due to hemolytic anemia with unclear details. ‡According to the IRIN press report. ††Based on IDSR data accessed via Hoff (2014) during the peak of reporting, only 26.5% of health zones (HZ) were reporting monkeypox cases. ‡‡The most current and rectified data employed for this outbreak. §Assumed no fatalities. §§From a total of 344 cases identified in the Katako-Kombe Health Zone (HZ). ‖IFRC values were utilized ‖‖The nine-month-old girl had a co-infection with *Plasmodium falciparum*. ¶ In 2016, Nolen et al. [21] reported 104 suspect cases. Five VZV positive cases were subtracted from the total, and five negatives for MPVX and VZV were included. ¶¶Referenced in Berthet et al. [15] **Monkeypox became a reportable disease to the IDSR in DRC in 2000. The death of the index case was reported, while the deaths of others went unreported. IDSR: integrated disease surveillance and response, IFRC: International Federation of Red Cross and Red Crescent Societies, IRIN: Integrated Regional Information Network.

Country	Democratic Republic of the Cong (DRC) (Zaire 1971–1997)*	South Sudan (Sudan pre-2011)	Gabon	Cameroon	Central African Republic (CAR)	Republic of the Congo (ROC)	Sierra Leone	Cote d’Ivoire	Nigeria	Liberia
1970–1974	1970: 1 (1) 100% [2], 1971: 0, 1972: 5, 1973: 3, 1974: 1						1970–71: 1 (1) 0% [3]	10/1971: 1 (U) 0% [3]	1971: 2 (1) 0% [3]	1970–1971: 4 (1) 0% [3]
1975–1979	1975: 3, 1976: 5, 1977: 6, 1978: 12, 1979: 8			1979: 2 (U) U% [4]					1978: 1 (1) 0% [3]	
1980–1984	1980: 4, 1981: 7, 1982: 40, 1983: 84, 1984: 86		1987: Lambarene: 1 (1) 100%^‖ ‖^ [5]		1984: 6 (U) U% [4]			1981: 1 (U) U% [4]		
1985–1989	1985: 62, 1986: 59 [4], 1981–1986: 338 (U) 9.8% [4]			1989: 1 (1) 0% [6]						
1990–1994			01/1991–06/1991: Region between Lamberene and N’Djole: 9 (5) 0% [7]							
1995–1999	02/1996–02/1997: Katako-Kombe HZ: 92 (12) 3.3%^‡‡ ^[8] 02/1996– 10/1997: Kasai oriental: 511 (U) (1.5%)^ §§^ [9] 03/1997–05/1997: Katako-Kombe n = 112, Lodja Nord n = 58, Sud HZ n = 11: 170 (U) 0% (weekly epidemiological record) [10]									
2000** –2004	02/2001–08/2001: Equateur Province: 23 (9) 21.7% [11] 2001: 388 (U) n = 13 (3.4%) 2002: 881 (1.6%) 2003: 755 (2.1%) 2004: 1024 (2.8%)				14/08/2001: Pimu CAR/DRC border: eight cases in a family (U) 25% [12]^¶¶^	15/04/03– 23/06/2003: Likouala department: 12 (3) 8.3%† [13]				
2005–2009	2005: 1708 (1.5%), 2006: 783 (2.6%), 2005-2007: Sankuru District: 1407 (703) U% [13], 2007: 970 (1.1%), 2008: 1599 (4.2%), 2009: 1919 (1.4%)	10/2005: Unity State: 37 (10) 0% [14]				2007: Likouala department: 62–150+ (U) U%‡ (IRIN, Reliefweb) [15]				
2010–2014	2010: 2322 (1.1%), 2011: 2208 (0.7%), 2012: 2629 (1.3%), 2013: 2460 (1.5%)†† (IDSR data) [16], 2013: Bokungu HZ: 99 (50) 10 (10.1%) [17]^¶^				06/2010: Deep forest, Southern CAR, 480 km from DRC border: 2 (2) 0%^§ ^[11] 30/04/2012: Batangafo: 2 (U) U% [11]	04/10–11/2010: Likouala department: 11 (2) 9.1% [18]	2014: Bo District: 1 (U) U% [19]			
2015–2018	1/1/2016–1/3/2016: Ateki HZ: 155 (7) 11 (7.1%) [21], 01/01/2018– 08/07/2018: IDSR: 2995 (U) 1.2% [22]			30/04/2018: Njikwa Health District: 6 (1) 0% [23]	30/01/2015: Bria: 3 (U) n = 1 33% 04/12/2015-02/2016: Bakouma and Bangassou subprefectures, Mbomou province: 10 (3) 20% [24], 04/12/2015–28/09/2016: Additional three cases reported^‖^ (IFRC) [25], 04/09/16–07/10/2016: Haute-Kotto health district: 26 (4)	18/01/17– 15/10/17: Likouala department: 88 (7) 6.8% [29]	14/03/2017: Pujehun district: 1 (1) 0% [20]		2017–18: 24 States: 228 (89) 2.6% [30]	12/2016 1 (U) U% [31]

Outbreak Investigation of Monkeypox Virus Infection Between 2018 and 2022

In Table 2, human MPVX outbreaks in Congo showed the urgent need to increase surveillance and preparedness capacity to prevent and control the outbreaks, while the emerging trends of MPVX infections in South Africa suggested the need for a One Health approach for disease detection as well as wildlife surveillance and investigations into the animal reservoirs.

**Table 2:** Summary of study findings on monkeypox virus infection outbreaks between 2018 and 2022. MPVX: Human monkeypox virus.

Author; published year	Objective	Study design	Study population	Outcomes
Najeeb et al., 2022	Monkeypox virus: A spreading threat for Pakistan	Review analysis	Pakistan	Despite public health experts declaring monkeypox a ‘containable disease’ (unlike COVID-19), the Pakistani government and health ministries must take this matter seriously and adequately prepare for a potential outbreak.
Sklenovská et al., 2018	Trace all reported human monkeypox outbreaks and relevant epidemiological information	Systematic review analysis	Congo	Urgent need to focus on building surveillance capacities that will provide valuable information for designing appropriate prevention, preparedness, and response activities.
Durski et al., 2018	MPVX virus as an emerging trend in humans	Review analysis	South Africa	As with all zoonotic diseases, a comprehensive One Health approach is necessary for disease detection and response, including wildlife surveillance and investigations into the animal reservoir/reservoirs, which require dedicated resources.
Diaz et al., 2021	Zoonotic orthopoxvirus outbreaks	Review analysis	Middle East and India, buffalopox in India, vaccinia in South America, and novel emerging orthopoxvirus infections in the United States, Europe, Asia, and South America	Outbreaks have occurred repeatedly worldwide

Mode of Transmission and Risk Factors

In the DRC, sleeping on the same floor as the animals was significantly (odds ratio (OR) 6.1, 95% confidence interval (CI) 1.2-31.6) associated with contracting MPVX, while eating or processing animal foods was not a significant risk factor. In the United States, cleaning the cages and bedding of a sick animal (OR 5.3, 95% CI 1.4-20.7), touching an infected animal (OR 4.0, 95% CI 1.2-13.4), or daily direct or indirect exposure to sick animals (OR 4.0, 95% CI 1.2-13.4) were associated with an increased probability of contracting MPVX infection (Table 3).

**Table 3:** Summary of study findings on risk factors for the primary introduction of monkeypox. NA: not applicable, ND: not described. *Conference abstract, no methodology provided. †Three controls were found to have elevated levels of IgM to orthopoxvirus.

Country	Risk factor	OR	aOR for vaccination status (95% CI)	P-value	Number of cases, n (number of controls, n)
Democratic Republic of the Cong (DRC) [2]*	Vaccinated	0.1 (0.03, 0.6)	NA	ND	252 (653)
	Exposure to Gambian rats	ND	2.6 (1.6, 4.1)	ND	
	Large terrestrial rodents	ND	1.8 (1.1, 3.0)	ND	
	Prosimians	ND	1.9 (1.2, 2.8)	ND	
	Non-human primates	ND	2.7 (1.4, 4.9)	ND	
Democratic Republic of the Cong [DRC] [3]	Live in a house with a door	0.07 (0.01, 0.6)	ND	0.01	15 (50)
	Prepared wild animals for consumption	0.2 (0.09, 0.1)	ND	0.04	
	Ate duiker	0.15 (0.03, 0.7)	ND	0.01	
	Sleep on floor	6.1 (1.2, 31.6)	ND	0.03	
USA [4]	Vaccinated	0.3 (0.1, 0.9)	NA	ND	30 (35)^†^
	Touched an infected animal	3.8 (1.2–11.7)	4.0 (1.2, 13.4)	ND	
	Cleaned the cage or touched the bedding of an infected animal	5.3 (1.5, 18.9)	5.3 (1.4, 20.7)	ND	
	Received scratch from an infected animal	5.6 (1.1, 28.6)	3.9 (0.7, 21.1)	ND	
	Daily indirect or direct exposure to an ill animal was significantly associated with contracting a monkeypox virus infection	3.8 (1.2, 11.7)	4.0 (1.2, 13.4)	ND	
	Having come within six feet, but not touched an infected animal	2.0 (0.6, 6.2)	2.0 (0.6, 6.5)	ND	

Discussion

This systematic review provides a comprehensive assessment of the epidemiologic evolution of MPVX infection since its first detection among humans in the 1970s. We found a greater than 14-fold increase in confirmed MPVX cases over the past five decades, from 55 cases in the 1970s to 793 cases in the 1990s, with the DRC being the most affected country. As a result of the recent outbreak, the number of confirmed cases in Nigeria has escalated from three cases in the 1970s to 341 cases between 2015 and 2018 [1-3]. There is a growing concern regarding the geographical spread and re-emergence of MPVX. In the last five decades, MPVX outbreaks have been reported in 10 African countries [1-3]. Concerningly, the re-emergence of MPVX occurred in Nigeria, Liberia, and Sierra Leone after 40 years, and in the CAR after 30 years. We report a rapid rise of MPVX cases in the highly endemic region of the DRC after the 1970s, which is also spreading to other countries. The cessation of the smallpox vaccination might be one of the factors responsible for these findings. 

Monkeypox typically transmits to humans from wild animals, such as rodents and primates (animals-to-humans), or, in some cases, from one infected individual to another (humans-to-humans) [4,36-38]. Nine studies showed that the source of infection was sexual contact, especially with male partners. Six studies mentioned that the cause of infection was contact with an individual with monkeypox symptoms. Two studies considered cases based on acquired nosocomial infections. Ingestion of barbecued bushmeat was the source of infection in three studies, while rodent carcasses were the source of infection in two other studies [39-42]. The opportunistic infection predominantly spreads through contact, such as hunting or consuming disease-ridden animals [4,36]. It can also spread through large aerosol droplets or contact with body fluids (saliva and blood), infected skin lesions, or contaminated materials, such as the clothing of an infected individual [4,37]. Following an incubation period of 5-21 days, the infection clinically manifests as fever, headache, myalgia, lymphadenopathy, and fatigue, similar to the presentation of the smallpox infection [4,37]. Individuals infected with a more severe form of the disease may also develop a characteristic ‘monkeypox rash,’ starting from their face and hands and later spreading to other body parts [5,38]. The lesions, which typically start as macules, progress into papules, followed by fluid-filled vesicles, pus-filled pustules, and subsequently, scabs that crust and eventually fall off [5,38]. The disease is often self-limiting, with the symptoms eventually resolving within two to four weeks. As supported by historical data, the smallpox vaccine (Imvanex) is 85% effective against monkeypox. Similarly, antivirals, including tecovirimat, cidofovir, and vaccinia immune globulin intravenous (VIGIV) approved for use against smallpox infection, can also be used for treating monkeypox infections [36,37]. 

Due to the atypical disease presentation and transmission patterns, the recent MPVX surge is rare and unusual compared to earlier outbreaks [36,37]. As reported by health experts, most of the monkeypox cases recorded in the past month in the western hemisphere have no known epidemiological links to endemic countries [37]. Additionally, most of the cases were documented among young men engaging in homosexual or bisexual activities, commonly known as “men who have sex with men” (MSM) [37]. Most of these cases presented with genital or peri-genital lesions, indicating a new route of disease spread-sexual contact. Although a definite cause for the sudden upsurge in cases is yet to be determined, health experts worldwide have suggested several theories that are currently under investigation. As of 2022, the genomic sequences of ten MPVX strains associated with the recent countrywide outbreak have been determined. While the West African Clade is mostly implicated in the recent viral surge, the data were insufficient to determine which mutation contributed to the increased transmissibility [38,39]. We found that in the DRC, sleeping on the floor was significantly associated with contracting MPVX, while eating or processing animal foods was not a significant risk factor. In the United States, cleaning the cages and bedding of sick animals, touching infected animals, and daily exposure to sick animals were associated with an increased probability of contracting MPVX infection.

Health officials stopped recommending smallpox inoculation following the eradication of smallpox in the late nineties [36,37]. Given that smallpox and Monkeypox viruses belong to the same genus (Orthopoxvirus), the cessation of the smallpox vaccine resulted in populations developing a weakened immunity to MPVX [37]. The waning immunity against the virus over several years served as a nidus for infection, resulting in the latest resurgence. Almost five decades ago, MPVX was considered a “disease of young children,” predominately affecting children aged four to five years [37]. By the 2000s, this number had increased five times, with 20-25-year-old individuals being most vulnerable to infection [38,39]. This is in line with the current data, which suggests that most of the recently reported MPVX cases have been among young men. It is highly likely that most of these individuals were not administered the smallpox vaccine either because they were too young or were born after the termination of routine vaccination.

The resumption of everyday life following the COVID-19 pandemic can also be attributed as a factor favoring the spread of MPVX. With the lifting of travel restrictions and the easing of social distancing measures, individuals were more likely to have been in close physical contact, thereby encouraging viral transmission. Moreover, the decline in the number of dendritic cells, and consequently the wearying of the immune response post-COVID-19 infection could have made individuals who had recovered from COVID-19 more vulnerable to secondary infections, and the resurgence of monkeypox might just be another testament to this fact.

## Conclusions

Recent global outbreaks and the rising incidence of MPVX infections among young adults in the endemic zones might be a result of the cessation of the smallpox vaccine. The increased risk associated with exposure to sick animals or sleeping on the floor suggests high infectivity from animal excretions. Increasing awareness, strict surveillance, and contact tracing can help contain global outbreaks. The ring vaccination approach for exposed individuals is another potential disease containment strategy. Future studies should investigate measures for rapid laboratory diagnosis, maintaining lab safety, and transmissibility. In addition, given the fact that diagnosing MPVX infections based on their symptoms is not a reliable approach, it is imperative that the WHO urgently procures the required testing kits, primers, and reagents to test for and combat a possible viral epidemic.

Raising awareness among healthcare providers and the general population, implementing strict surveillance measures, and timely contact tracing (in case of a viral outbreak) are the best preventative measures to effectively prevent or manage a potential monkeypox outbreak in the country. Per expert-issued guidelines, mass smallpox vaccination campaigns are not a requirement. Instead, a ring vaccination approach needs to be utilized, which involves the vaccination of only individuals who have been in close contact with infected individuals. This way, the challenges presented due to the limited global supply of smallpox vaccine and the prevalent vaccination hesitancy in the world can be overcome, albeit to some extent.
